# A guide to characterizing the dynamic mitochondria–endoplasmic reticulum contact sites

**DOI:** 10.1111/febs.70184

**Published:** 2025-07-07

**Authors:** Antigoni Diokmetzidou, Luca Scorrano

**Affiliations:** ^1^ Department of Biology University of Padova Italy; ^2^ Veneto Institute of Molecular Medicine (VIMM) Padova Italy

**Keywords:** endoplasmic reticulum, imaging, membrane contact sites, mitochondria, mitochondria–ER contact sites

## Abstract

Organelles were once regarded as discrete entities, but it is now established that they interact through specialized membrane contacts maintained by protein tethers and lipid interactions. Among these, mitochondria–endoplasmic reticulum contact sites (MERCS) emerged as hubs for calcium signaling, lipid metabolism, and mitochondrial dynamics. Here, we critically appraise current methodologies for MERC visualization and quantification, survey the molecular toolbox for their selective perturbation, and highlight common experimental pitfalls. We also discuss key conceptual issues—defining MERCs on structural and functional grounds, addressing redundancy among tethering factors, and distinguishing primary MERC‐mediated effects from secondary cellular responses. Finally, we propose that an integrative strategy combining imaging, precise biochemical isolation, proteomics, and functional assays will be essential to resolve outstanding questions about MERC dynamics in physiology and pathology.

## Graphical abstract

The dynamism, heterogeneity, and functional complexity of mitochondria–endoplasmic reticulum contacts (MERCs) require combined methodological approaches to offset limitations of individual techniques. Here, we review available tools and methodologies for MERC research, outline key technical challenges, and discuss how recent advances deliver unprecedented spatiotemporal resolution of MERC dynamics, opening new research avenues.

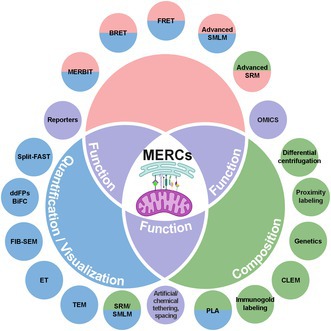

**Abbreviations**
AI: artificial intelligence
AKAP1: A‐kinase anchoring protein 1
APEX: ascorbate peroxidase‐based proximity labeling
BiFC: bimolecular fluorescence complementation
BRET: bioluminescence resonance energy transfer
CFM‐SCT: cryogenic fluorescence microscopy and soft X‐ray tomography
CFP: cyan fluorescent protein
ChiMERA: construct helping in mitochondria‐ER association
CLEM: correlative light and electron microscopy
CRISPR: clustered regularly interspaced short palindromic repeats
CsFiND: complementation assay using fusion of split‐GFP and TurboID
EM: electron microscopy
EMC: ER membrane complex
ER: endoplasmic reticulum
ERMES: ER‐mitochondria encounter structure
ET: electron tomography
FABCON: fluorogen‐activated bimolecular complementation at contact sites
FACT: fluorescence‐assisted diffraction computational tomography
FATE1: fetal and adult testis expressed 1
FEMP: FRET‐based indicator of ER–mitochondria proximity
FFAT: two phenylalanines in an acidic tract
FIB: focused ion beam
FKBP: FK506‐binding protein
FP: fluorescent protein
FRB: FKBP–rapamycin binding domain
FRET: Förster Resonance Energy Transfer
GFP: green fluorescent protein
GI‐SIM: grazing incidence structured illumination microscopy
IP3: inositol 1,4,5‐trisphosphate
ISACS: *in situ* analysis of contact sites
ISM: image scanning microscopy
LIT: light‐induced tethering
LOV2: light‐oxygen‐voltage 2 domain
MAMs: mitochondria‐associated membranes
MCS: membrane contact sites
MERBiT: mitochondria endoplasmic reticulum contact reporter applying NanoBiT
MERCS: mitochondria‐ER contact sites
MERCs: mitochondria‐ER contacts
MERLIN: mitochondria‐ER length indicator nanosensors
MFN2: mitofusin 2
MOC: Manders' overlap coefficient
MoNALISA: molecular nanoscale live imaging with sectioning ability
MSI: multispectral imaging
OMM: outer mitochondrial membrane
PAINT: point accumulation for imaging in nanoscale topography
PALM: photoactivated localization microscopy
PCC: Pearson's correlation coefficient
PDZD8: PDZ domain‐containing protein 8
PERK: Protein kinase R (PKR)‐like ER kinase
PLA: proximity ligation assay
PRINCESS: probe for interorganelle Ca^2+^‐exchange sites based on SplitFAST
RESOLFT: reversible saturable optical fluorescence transitions
SEM: scanning electron microscopy
SIM: structured illumination microscopy
SMLM: single‐molecule localization microscopy
SPLICS: split‐GFP‐based contact site sensor
SplitFAST: split fluorescence‐activating and absorption shifting tag
SR: super‐resolution
SRM: super‐resolution microscopy
STED: stimulated emission depletion microscopy
STORM: stochastic optical reconstruction microscopy
SXT: soft X‐ray tomography
TEM: transmission electron microscopy
VAP: vesicle‐associated membrane protein‐associated protein
VAPB: vesicle‐associated membrane protein‐associated protein B
VDAC1: voltage‐dependent anion channel 1
YFP: yellow fluorescent protein

## Introduction

The concept that organelles are physically isolated entities has been abandoned. We now understand that they engage in juxtaposition through close membrane contacts maintained by protein tethers or protein–lipid interactions, without undergoing membrane fusion. These contact sites form specialized subdomains, each defined by a unique composition and function, which enable direct communication and coordination between organelles. Among these, mitochondria–endoplasmic reticulum contact sites (MERCS), where the mitochondrial outer membrane is juxtaposed to the endoplasmic reticulum (ER) membrane, are the most extensively characterized. Indeed, as early as the 1950s, transmission electron microscopy (TEM) provided the first structural insights into these regions [1, 2]. Subsequently, TEM coupled with differential centrifugation allowed isolation and biochemical characterization of the fractions now known as mitochondria‐associated membranes (MAMs), and pioneering studies then unveiled their molecular composition and functional roles [3, 4].

**Fig. 1:**
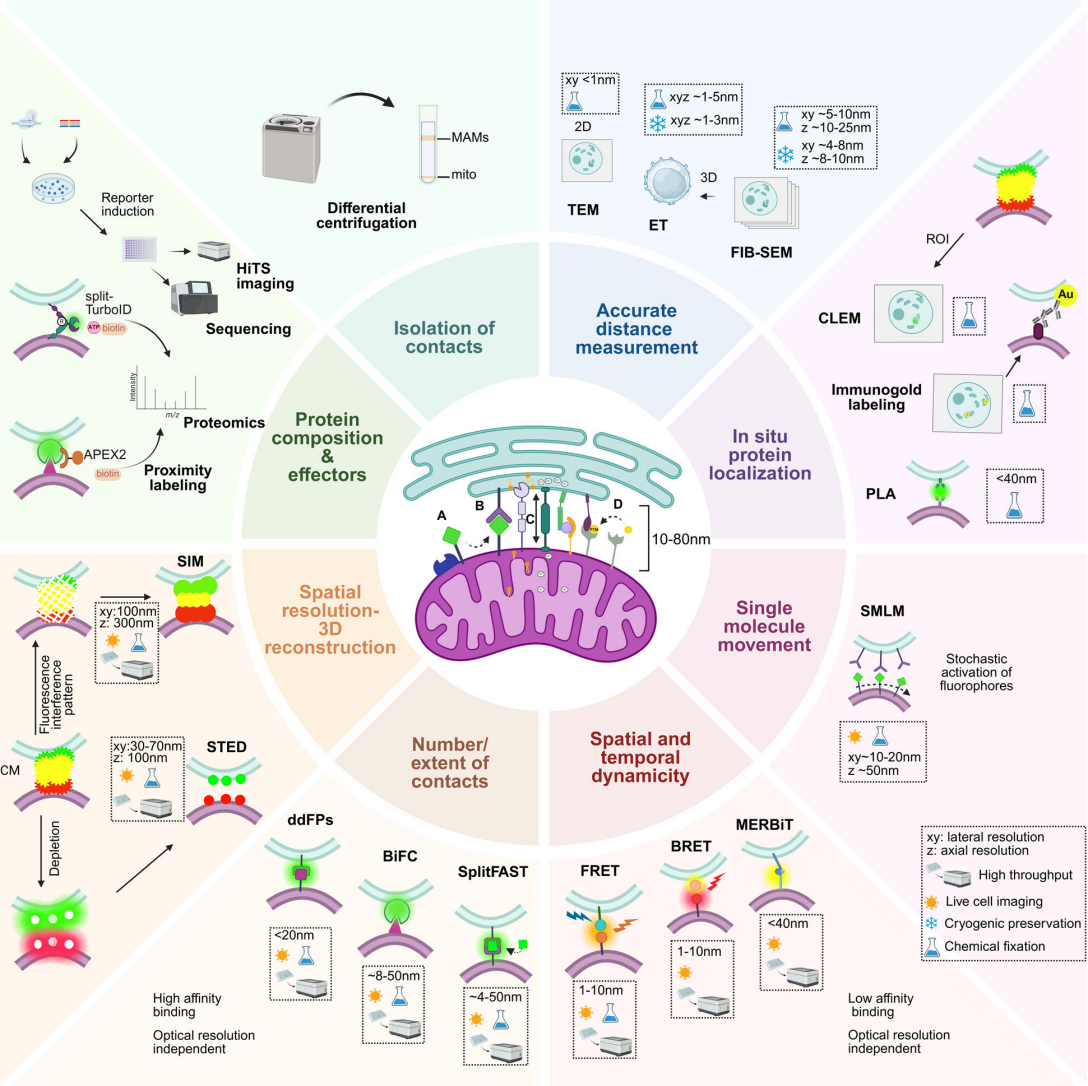
Overview of mitochondria‐ER contact sites (MERCs) characterization. Center: a schematic depiction of MERCs showing the physical interface between mitochondria and ER. The key categories of proteins residing in MERCs are indicated: (A) sorter/recruitment proteins, (B) tethers, (C) functional and (D) regulators. Crown: a summary of the imaging, including electron microscopy and super‐resolution microscopy, and non‐imaging techniques such as proximity‐based assays used to study MERCs.

Although progress in the field was initially gradual, the development of innovative techniques and the continual expansion of our experimental toolbox have exponentially accelerated our understanding of MERCS. Electron microscopy and subcellular fractionation remain cornerstone approaches, now enriched by a diverse array of methods to visualize MERCS, interrogate their molecular composition, and probe their functions (Fig. 1), revealing a previously unappreciated level of spatial and temporal dynamism. Here, we offer a concise overview of the current tools and methodologies, and we highlight key considerations for rigorously studying MERCS.

## Visualization and quantification of MERCs by imaging

### Diffraction‐limited light microscopy

The intermembrane gap at MERCs varies from ~10 to 80 nm, both among different cell types and within individual cells. For example, the mitochondria–smooth ER separation in RBL‐2H3 cells is of ~25 nm [5], whereas in HT‐1080 cells it measures ~15 nm [6]. In hepatocytes, the rough ER‐associated mitochondria maintain a ~54 nm distance from the ribosomes of the rough ER [7], similar to the 50–60 nm distance in HT‐1080 cells [6], while in COS‐7 cells, rough ER–mitochondria contacts span ~35 nm [8]. These distances lie well below the optical diffraction limit (~250 nm), precluding direct visualization by standard widefield or confocal microscopy. Nonetheless, diffraction‐limited approaches remain useful for live or fixed specimens when interpreted cautiously. Image scanning microscopy (ISM), such as Airyscan, doubles confocal resolution (lateral: from ~250 nm to ~120 nm; axial: from ~600–700 nm to ~350 nm), allowing relative measurements of pseudocolocalization of fluorescent organelle markers or of immunofluorescently labeled proteins at MERCs, albeit with potential errors of several hundred nanometers. Moreover, widefield and confocal systems readily detect signals from proximity‐based reporters, and the integration of multispectral imaging (MSI) enhances contrast and specificity. Indeed, MSI allowed the simultaneous visualization of multiple organelles in living cells, uncovering their mutual interactions [9] and the coordination of multiorganellar pathways [10].

### Electron microscopy

Transmission electron microscopy (TEM) was instrumental to first reveal the existence of MERCs in the 1950s [1], and it has been since then used to generate two‐dimensional images from ultrathin slices with sub‐nanometer resolution [11, 12, 13]. Despite its spatial fidelity, TEM's low throughput, lengthy sample preparation, and limited field of view complicate the detection of infrequent or subtle alterations (see Table 1). Correlative light and electron microscopy (CLEM) mitigates these limitations by localizing regions of interest via light microscopy before ultrastructural TEM imaging. Immunogold labeling further permits precise localization of MERC proteins [14, 15, 16].

**Table 1:** Pros and cons of imaging techniques for MERCs.

Technique	Advantages	Disadvantages	References
Electron microscopy‐based			
TEM	High spatial resolution	2D approach. Thin sections are required. No information on temporal dynamicity. Requires dehydration and staining	[5, 18, 140]
SEM	3D reconstruction is possible High spatial resolution	Lower resolution compared to TEM or ET. Requires dehydration and staining. Requires serial sectioning. Computationally intensive	
ET	High‐resolution 3D reconstruction Very high resolution	Limited range of tilting. Requires serial sectioning for the construction of the 3D model. Requires chemical fixation, dehydration and heavy metal staining. Computational heavy	[19]
Cryo‐ET	MERCs are preserved in native conditions Near‐atomic resolution Doesn't require chemical fixation or heavy metal staining Allows visualization of dynamic interactions	Thin sections required‐sample's thickness remains below 100–200 nm. Requires dedicated high‐end microscopes and technical expertise	[22, 25, 26]
FIB‐SEM	Wider range of sample thicknesses compared to TEM or ET Suitable for the study of large‐volume MCSs within the interior of cells	Requires dedicated high‐end microscopes and technical expertise. Computationally intensive	[141, 142, 143]
Cryo‐FIB‐SEM	Direct visualization of MERCs within the intact cellular context	Requires specialized equipment and Expertise. Computationally intensive	[22]
STX	3D reconstruction High‐resolution imaging of internal structures Does not require chemical staining	Requires specialized synchrotron radiation sources and high‐end imaging equipment. The natural electron density differences may limit the distinguishability between certain structures	[46]
Super‐resolution fluorescence microscopy‐based			
SIM	High sensitivity Use of common fluorophores. 3D reconstruction. Fast imaging speed and low excitation intensity. High‐throughput use. Live‐cell nanoscale imaging	Limited spatial resolution (lateral: 100 nm, axial: 300 nm). Requires dedicated high‐end microscopes and technical expertise	[34, 144]
GI‐SIM	High signal‐to‐noise ratio compared to conventional widefield 2D SIM. Better 3D reconstruction. High spatial (axial:100 nm) and temporal resolution. Live‐cell imaging with minimal phototoxicity. Allows millisecond‐scale resolution	Requires dedicated high‐end microscopes and technical expertise	[39, 40]
TIRFM	High axial resolution (100 nm) Use of common fluorophores	Only images close to PM. Limited lateral resolution (200 nm). Requires dedicated high‐end microscopes and technical expertise	[22]
STED	High lateral (30 nm) and axial (100 nm) resolutions. Allows 3D reconstruction	Limited multi‐color imaging. Requires photostable fluorophores, high‐power laser and longer image acquisition time that causes phototoxicity and limited temporal resolution. Requires dedicated high‐end microscopes and technical expertise	[8, 33, 41]
RESOLFT	Large field of view (50 × 50 μm^2^). ~45–65 nm spatial resolution. Suitable for both rapid and prolonged time‐lapse recordings. Low light intensities (W‐kWcm^−2^). Reduced phototoxicity	Requires dedicated high‐end microscopes, technical expertise, advanced computational resources and specialized software	[43]
SMLM (STORM/PALM)	Very high lateral (~10–20 nm) and axial resolutions (50 nm)	Limited 3D, multi‐color imaging, and temporal resolution. Requires photostable fluorophores, dedicated high‐end microscopes, technical expertise, advanced computational resources and specialized software	[36, 113, 145]
SR‐FACT	High resolution and sensitivity. Fast imaging for dynamic changes. 3D reconstruction. Live‐cell imaging. Long‐lasting time courses. Unlabeled imaging of organelles	Requires dedicated high‐end microscopes, technical expertise, advanced computational resources and specialized software	[45]
CFM‐SXT	Simultaneously imaging of multiple compartments. High resolution. Inherent organelle specificity based on 2D/3D morphology and absorption contrast. No additional staining to enhance contrast	Requires high‐end microscopes and soft X‐ray illumination, advanced computational resources and specialized software	[46, 146]
Proximity‐based probes			
FRET	High distance sensitivity. Spatial resolution of 1–10 nm. Live‐cell imaging. It can be combined with different techniques. Does not require special equipment. High throughput. Does not induce artificial contacts	Orientation, equal expression and spectral overlap dependent. Photobleaching of the donor over time. Its range depends on linker sequences, otherwise limited to 1–10 nm distance. The signal detected depends on the distance rather than the detection of individual contacts	[12, 19]
BRET	Less phototoxicity compared to FRET. Does not require exogenous illumination. Higher sensitivity compared to FRET. Does not induce artificial contacts	Requires the addition of luciferase substrate. The activity of luciferase depends on environmental factors like pH, ATP levels. Its range depends on the linker sequence (MERLIN), otherwise it is limited to 1‐10 nm distance. The signal detected depends on the distance rather than the detection of individual contacts	[50, 51]
BiFC	Easily quantifiable fluorescence signals. High throughput	Irreversible complementation. overexpression of Can induce artificial contacts and distort organelle morphology. Complementation depends on the orientation and positioning of the fused FP fragments. High local concentrations can induce self‐assembly	[57, 58, 60, 61, 64]
SplitFAST	Reversible. Fast complementation. Low self‐complementation. No fluorescence leakiness. Live‐cell imaging. Improved brightness with splitFAST2. High throughput. High temporal resolution	The detected contacts depend on the distance of the linkers inserted into the probe	[68, 69]
PLA	Detection of endogenous proteins and low abundant proteins. Easy to use	Depends on antibody availability and specificity. Fixed samples	[143, 147]
ddFP	Reversibility. Simultaneous detection of two different MCS sharing the same organelle (Contact‐FP toolkit)	Limited brightness. Low contrast and sensitivity	[75, 76, 77]

Electron tomography (ET) extends TEM into three dimensions, enabling high‐resolution reconstructions that were used to demonstrate the first physical tether at MERCs [5] and to validate the tethering role of specific proteins [17, 18, 19]. ET also provided direct evidence that ER governs mitochondrial division sites [20] and the distribution of mitochondrial nucleoids [21]. Cryo‐ET, which preserves samples in vitrified ice at cryogenic temperatures, achieves near‐atomic resolution of MERC‐resident complexes *in situ* [22, 23], elucidating, for example, the flexibility of VAP‐A in forming 10–30 nm contacts [24, 25] and the lipid‐transfer architecture of VPS13 [26].

Focused ion beam scanning EM (FIB‐SEM) generates volumetric tomograms by alternately slicing and imaging the specimen surface. Coupled with deep learning–based segmentation, FIB‐SEM now delivers high‐resolution, high‐throughput imaging of extensive tissue volumes [27, 28, 29]. Deep learning pipelines have similarly accelerated automated analysis of TEM and volume EM datasets [30, 31]. Nevertheless, 3D EM approaches remain laborious, computationally intensive, and unsuited to live‐cell dynamics, with time‐resolved cryo‐EM still facing formidable technical hurdles.

### Super‐resolution microscopy

Super‐resolution microscopy (SRM) overcomes the diffraction barrier, achieving ~20 nm spatial resolution and suitable temporal performance in live and fixed samples. Techniques such as structured illumination microscopy (SIM), stimulated emission depletion (STED), and single‐molecule localization microscopy (SMLM) (Fig. 1) have all illuminated MERC architecture and dynamics [32, 33, 34, 35, 36, 37, 38]. Grazing incidence SIM (GI‐SIM) captures rapid organelle movements and cytoskeletal interplay in real time [39]. GI‐SIM was also used to achieve super‐resolution live‐cell imaging of the ER‐late endosome–mitochondria contact sites mediated by PDZD8 [40]. Live‐cell dual‐color STED increases the axial resolution of STED and mitigates the phototoxicity arising from the high power of the depletion beam while tracking interactions between SNAP‐tagged mitochondria and Halo‐tagged ER [33, 41]. Advanced implementations—multi‐color STED, RESOLFT (e.g., MoNALISA [42])—have visualized ER tubule dynamics around mitochondria in neurons at ~50 nm resolution over seconds to minutes [43, 44].

SMLM modalities such as photoactivated localization microscopy (PALM), stochastic optical reconstruction microscopy (STORM), and points accumulation for imaging in nanoscale topography (PAINT) offer the highest resolution but require specialized fluorophores and often struggle with fast dynamics due to slow acquisition and potential photobleaching. Hybrid methods such as super‐resolution fluorescence‐assisted diffraction computational tomography (SR‐FACT) [45] and correlative cryogenic fluorescence microscopy with soft X‐ray tomography (CFM‐SXT) [46] reconstruct 3D nanoscale architecture in whole cells without sectioning, albeit demanding top‐tier instrumentation and expertise.

### Proximity‐based methods

Proximity assays sensitively detect membrane apposition in live cells, though they do not resolve fine architecture. Fluorescence resonance energy transfer (FRET) monitors energy transfer at <10 nm separations and has been adapted into probes such as FEMP (FRET‐based indicator of ER–mitochondria proximity) to report dynamic MERC changes via a self‐cleaving Tav2A linker that ensures equimolar donor–acceptor expression [12, 19, 47, 48]. Bioluminescence resonance energy transfer (BRET) variants, including the mitochondria‐ER length indicator nanosensors MERLIN and the MAM‐specific BRET‐based Ca^2+^ indicator MAM‐Calflux, achieve reduced phototoxicity but depend on substrate addition and are poised to signal variability and sensitivity to environmental conditions. Both FRET and BRET‐based probes have the disadvantage that they cannot be applied for the simultaneous detection of contacts at different distances (see Fig. 1 and Table 1). Moreover, since they are proximity‐based techniques, they cannot ensure that the recorded signal arises from physical bridges between the organelles; rather, it depends on their close apposition [49, 50, 51].

Bimolecular fluorescence complementation (BiFC) sensors [52] like superfolded GFP [53, 54], split‐GFP, ‐Venus, ‐YFP [55, 56, 57, 58], the split‐GFP‐based contact site sensor SPLICS [59, 60] or inducible systems [61] map MERC distribution and dynamics under physiological or stress conditions [57, 62, 63] and have been used to discover new tethers [64]. However, their irreversible reconstitution can yield false positives or perturb organelle morphology [65, 66]. SplitFAST probes introduce reversible complementation with exogenous fluorogenic dyes [67]. FABCON (fluorogen‐activated bimolecular complementation at contact sites) was the first splitFAST probe reported to detect MERCs and other organelle contact sites [68]. The development of short and long ER‐mitochondria splitFAST probes allowed the detection of MERCs with distances less than 12 nm or 25 nm [69]. Integration of Ca^2+^‐sensing capabilities to splitFAST generated PRINCESS (probe for interorganelle Ca^2+^‐exchange sites based on splitFAST), a dual functionality probe like MAM‐CAlflux that can simultaneously detect MERCs and measure Ca^2+^ dynamics [69]. Split‐NanoBiT like MERBiT, the mitochondria endoplasmic reticulum contact reporter applying NanoBiT [70], or the SpLacZ reporter that utilizes α acceptor and α donor LacZ fragments targeted to the mitochondria and ER, enable live‐cell, reversible detection of contact formation [71]. Dimerization‐dependent fluorescent proteins (ddFPs) are based on the binding of two dark FP monomers that lead to reversible formation of a fluorescent heterodimeric complex [72]. They offer reversible reporting with low background and have therefore been used to detect MERCs and other MCS [73, 74], characterize tethers [19], and validate the colocalization and function of proteins at MERCs [75, 76, 77], albeit they are often limited by a low signal‐to‐noise ratio.

Finally, *in situ* proximity ligation assays (PLA) detect endogenous protein interactions (<40 nm) in fixed cells or tissues by using antibodies against MERC‐resident proteins, and via rolling‐circle amplification, most commonly probing VDAC1–IP_3_R pairings at MERCs [78, 79, 80]. However, the VDAC1–GRP75‐IP_3_R complex seems to functionally couple mitochondria and ER at ~20–30 nm that allows for effective Ca^2+^ exchange, and it can be used for probing MERCs dedicated to Ca^2+^ flux [81]. For the detection of MERCs participating in phospholipid biosynthesis and trafficking (usually of ~10 nm [81]), we advise using a different pair. PLA excels in fixed samples for quantifying contacts under physiological or pathological contexts without overexpression artifacts [82, 83, 84], but cannot capture live dynamics.

## Characterization of the MERC proteome and lipidome

A comprehensive delineation of the MERC proteome and lipidome is indispensable for elucidating the molecular tethers that bridge the two membranes and the signaling cascades located at the interface. The pioneering isolation of MAMs via differential centrifugation using a Percoll gradient first revealed the enrichment of phospholipid‐synthesizing enzymes within these domains [4]. A modified version uses sucrose gradient to isolate fractions enriched in wrappER‐associated mitochondria [85, 86]. This strategy and its modifications, applied across diverse cell types and tissues [87, 88, 89, 90] and combined with mass spectrometry–based proteomics [91, 92, 93], have cataloged MERC‐resident proteins. Nevertheless, bulk isolation cannot track MAM dynamics in real time nor capture fleeting compositional shifts.

To surmount these limitations, proximity‐dependent biotinylation approaches (BioID and APEX) have been used. BioID, employing the promiscuous biotin ligase BirA* to label proximal proteins, mapped interactors of the ER stress sensor PERK at MERCs, illuminating their roles in Ca^2+^ handling [94, 95]. The enhanced BioID2 variant, a smaller BirA* from *Aquifex aeolicus*, improves labeling efficiency and reduces the incubation time required [96]. Split‐BioID (“Contact‐ID”) further refined spatial specificity, identifying 115 MAM‐specific proteins, including FKBP8, the tethering partner of PDZD8 [97, 98] and trichoplein/mitostatin, a negative regulator of the tether Mfn2 [99]. More recently, TurboID and its split counterpart with accelerated labeling (less than 10 min) and increased specificity have been employed, enabling high‐resolution proteomic maps in mammalian cells [100, 101, 102]. In yeast, the CsFiND system (split‐GFP fused to split‐TurboID) visualized and identified ERMES components *in situ* [103]. APEX and APEX2 catalyze the rapid (<1 ms) oxidation of biotin‐phenol in the presence of H_2_O_2_, tagging amino acids within ~20 nm [104, 105]. These methods unveiled reticulon‐1A (RTN1A) [106] and the SYNJ2BP–RRBP1 tethering pair [107], though the requisite peroxide limits live‐cell applications due to oxidative stress. A bimolecular fluorescence complementation proximity‐labeling strategy (BiFCPL) integrating APEX2 identified 403 MERC proteins in a single experiment [108].

Beyond proteomics, genetic screens have proved instrumental: the ERMES complex emerged from a yeast mutant screen complemented by a synthetic ER–mitochondria linker [109], and a genome‐wide CRISPR/Cas9 screen in a split‐mVenus MERC reporter line revealed novel regulators, despite the low sensitivity of BiFC for loss‐of‐function hits [110].

## Manipulation of the mitochondria–ER Interface

Dissecting MERC function also requires tools to impose or perturb membrane apposition. Rapamycin‐induced dimerization of FKBP‐tagged mitochondria and FRB‐tagged ER (monitored by FRET) first demonstrated forced intermembrane tethering [12], later adapted in the FEMP probe to clarify Mitofusin 2 tethering role [19]. Though straightforward, this system lacks spatial precision and reversible control, and rapamycin itself, albeit applied only very briefly, can confound autophagy studies (see Table 1).

Optogenetic tethers offer refined spatiotemporal control. The light‐inducible dimer (iLID) system, in which LOV2‐caged SsrA and SspB bear OMM or ER localization signals respectively, enables blue‐light–triggered MERC formation with micrometer and second‐scale precision [111]. Similarly, engineered “eMags” magnets overcoming earlier concatemerization and stability issues afford rapid, reversible, light‐driven tethering with temporal and spatial fidelity [112].

Synthetic constitutive tethers have also evolved since the first mRFP‐bridged ER–mitochondria construct [5]. Helical EAAAR repeat linkers flank mRFP to span defined distances (<20 nm) [113]. One variant of these linkers expressed in mouse cardiomyocytes conferred stress resilience through adaptive remodeling [114]. A palette of helix‐based linkers spanning 5–30 nm in 5 nm increments [115] has been useful to reveal that a ~ 20 nm gap optimizes Ca^2+^ transfer and mitochondrial metabolism, due to local IP_3_R enrichment [113, 115]. The ChiMERA GFP‐based construct similarly was instrumental for the screening that led to the discovery of ERMES [109], and the Split‐MAM tool compensated for ERMES loss while modulating CoQ homeostasis [116]. Validation of candidate tethers via artificial linkers is powerful: rescue of a tether‐deficient phenotype strongly implicates the protein in membrane apposition, whereas lack of rescue, as in Mdm10‐null yeast, may reflect functional redundancy rather than absence of tethering capacity [109]. Moreover, the effective range of a synthetic linker must match endogenous contact distances; a 25 nm span may bridge hepatic rough ER–mitochondria contacts but fail to engage smooth ER sites in the same cell type or MERCs in other cell lines.

Endogenous tether modulation by overexpression or knockout of native tethers or spacers similarly alters MERC abundance and function. Ectopic FATE1 expression in hepatocytes uncouples mitochondria and ER, promoting insulin sensitivity and steatosis [117], while a corresponding artificial linker mitigates diet‐induced glucose intolerance [118]. Yet, endogenous proteins often possess non‐tethering roles: Mitofusin 2 ablation reduces MERCs but also fragments mitochondrial networks [18, 19, 119], necessitating orthogonal assays to specifically attribute phenotypes to MERC perturbation. Given the collective nature of tethering forces, loss of one component may diminish but not abolish contacts or specific functions, implying an auxiliary or condition‐specific tethering role [120].

Direct quantification of tethering forces remains challenging. Techniques such as laser scission [121], optical tweezers [122, 123], femtosecond laser ablation [124], and potentially atomic force microscopy [125] have measured forces at other organelle interfaces; more recently, *in situ* artificial contact sites (ISACS) between synthetic and endogenous membranes have quantified protein‐tethering activities via FFAT motif recruitment to VAPs [126].

Chemical modulation adds another layer of control [127]: small molecules can perturb MERC structure or function by targeting structural proteins [128, 129, 130], altering membrane lipid composition, modulating MERC protein expression [131], or engaging signaling and metabolic pathways that gate MERC stability [132, 133, 134, 135]. However, off‐target effects are common, and rigorous interpretation demands cognizance of each compound's specificity.

## Technical challenges

The diverse methodologies described herein each illuminate distinct facets of MERCs, yet no single approach is sufficient to capture their full complexity. MERCs are inherently four‐dimensional, ever‐shifting assemblies that rapidly form and dissolve in response to (patho)physiological signals as it was originally reported by rapid confocal imaging [136]. Thus, a single time‐point or single‐plane measurement offers only a snapshot of a highly dynamic interface.

Electron microscopy remains the benchmark for direct, nanometer‐scale measurement of intermembrane spacing, but it must be complemented by proximity‐based sensors or super‐resolution microscopy to map MERCs in both space and time. Obara *et al*. exemplify this integrative paradigm: combining FIB‐SEM structural reconstructions with live‐cell tracking of VAPB molecules, they revealed dynamic subdomains within MERCs distinguished by unique ER curvature profiles and rapid stimulus‐induced remodeling [36]. Likewise, parallel deployment of five cutting‐edge super‐resolution modalities uncovered the swift transition of ER from tight, tubular clusters to expansive networks that facilitate organellar interactions [137]. The molecular architecture of the yeast ERMES complex was resolved through an elegant fusion of quantitative live imaging, cryo‐CLEM, subtomogram averaging, and molecular modeling [22].

Downstream data analysis poses its own hurdles. Conventional colocalization metrics like Pearson's correlation and Manders' overlap coefficients are limited to ~200–300 nm resolution, risking overestimation of true contacts. Moreover, current interaction‐analysis algorithms cannot unlock the full potential of SRM methodologies, especially in multichannel settings. SRM requires extensive storage space and powerful computational algorithms customized to the data generated by each individual SRM technology. For example, point cloud‐based methods like SMLM are built on algorithms that analyze dense clouds of localization points to deduce spatial arrangements of labeled structures using clustering, nearest‐neighbor analysis, or probabilistic modeling. On the other hand, voxel‐based methods like STED or SIM produce image‐like data that are typically analyzed using correlation metrics [138], image segmentation, or intensity‐based proximity mapping. Due to diffraction artifacts, background noise, and signal heterogeneity, image segmentation is a challenging step for all pixel‐based microscopy methods attempting to quantify contact sites. Segmentation errors can affect spatial relationships and lead to misestimation of the contacts. Improving the resolution by deconvolution can further introduce artifacts, including edges or false positives at contact interfaces, especially when the imaging noise is amplified. The new analysis tools should integrate both spatial and temporal dimensions of cellular behavior and correct for modality‐specific artifacts like drift, anisotropy, and signal bleed‐through to ensure accurate contact mapping [139]. AI‐driven segmentation pipelines now overcome variable signal‐to‐noise ratios and subjective thresholding, enabling unbiased, high‐throughput quantification, 3D reconstructions, and live‐cell tracking. For instance, the MCS‐DETECT algorithm achieves subpixel resolution of volumetric fluorescence data to pinpoint MERCs beyond these conventional constraints [8]. Yet, many of these software solutions remain technically demanding and not user‐friendly.

Finally, the design and validation of novel probes or synthetic tethers require meticulous controls. Accurate targeting must be confirmed, and functional titration performed to prevent probe aggregation or structural perturbations. For example, as shown by the ChiMERA construct, swapping the yeast Tom70 presequence for the mouse AKAP1 targeting signal diminished tethering strength, underscoring the sensitivity of MERC mechanics to subcellular targeting sequences [109]. Moreover, metabolic changes and cellular stress can influence the composition of MERCs and the distance between organelles. We recommend that the experimental conditions be carefully chosen and maintained throughout the experimental campaign to avoid artifacts. Moreover, transfection‐based techniques are sensitive to changes in condition, further underlining the importance of constant, well‐controlled experimental conditions. Lastly, given that the extent and the types of MERCs differ among tissues and cell types, we advise carefully choosing the model where a specific MERC or MERC‐related function is studied.

## Conclusions

The remarkable heterogeneity of MERCs size, composition, and function defies any singular definition. Assigning “tether” status to a resident protein is equally elusive: rapid, signal‐dependent assembly, and disassembly of MERCs complicate functional attribution. Redundant networks of proteins often converge on the same processes, ensuring robustness but confounding the interpretation of loss‐of‐function experiments aimed at assessing if a protein works as a tether. Moreover, several MERC‐localized proteins moonlight elsewhere, so that perturbations may produce indirect, pleiotropic effects. Each technique discussed in this review displays advantages and shortcomings. To surmount the heterogeneity of MERCs and the limitations of each technique used to study them, we strongly advise a multifaceted approach that combines advanced imaging, precise biochemical isolation, and refined genetic and functional assays. Emerging technologies now offer unprecedented spatiotemporal resolution of MERC dynamics, promising to help address questions of mechanism and regulation under physiological and pathological conditions. Ultimately, a deeper mechanistic understanding of MERC architecture and signaling could not only tackle a key question in biology, but also help in the design of targeted therapeutics, harnessing these membrane contact sites to ameliorate disease.

## Conflicts of interest

The authors declare no conflict of interest.

## Author contributions

AD, LS: manuscript writing and revision; AD: visualization; LS: supervision, funding acquisition.
